# Maternal infection at delivery and risk of stillbirth and early neonatal death: nested case–control studies in East African pregnancy cohorts

**DOI:** 10.1186/s12884-026-09068-3

**Published:** 2026-04-20

**Authors:** Anna C Seale, Meron Kebede, Rosanna Glazik, Mussie Brhane, Mulu Berihun, Zelalem Teklemariam, Dadi Marami, Joseph Oundo, Tadesse Gure, Tseyon Tesfaye, Ann Karanu, Gumbi Wilson, Christina W. Obiero, Dianna M. Blau, Hellen Barsosio, Angela Koech, Samir Saha, Joy E. Lawn, Robert F. Breiman, N. Claire Gordon, Stefanie Wittmann, Lola Madrid, Abraham Aseffa, Yadeta Dessie, James A. Berkley, Nega Assefa, J. Anthony G. Scott

**Affiliations:** 1 Epidemiology and Population Health, London School of Hygiene & Tropical Medicine, London, UK; 2 College of Health and Medical Sciences, Haramaya University, Harar, Ethiopia; 3 KEMRI-Wellcome Trust Research Programme, Kilifi, Kenya; 4 Warwick Medical School, University of Warwick, Coventry, UK; 5 Haraghe Health Research Partnership, Harar, Ethiopia; 6 Centers for Disease Control and Prevention (CDC) Global Health Center, Atlanta, USA; 7 Centre of Excellence in Women and Child Health, Aga Khan University – Kenya, Nairobi, Kenya; 8 Armauer Hansen Research Unit, Addis Ababa, Ethiopia; 9 Child Health Research Foundation, Dhaka, Bangladesh; 10 Rollins School of Public Health, Emory University, Atlanta, USA; 11 Infectious Diseases and Oncology Research Institute, University of the Witwatersrand, Johannesburg, South Africa; 12 African Population and Health Research Center, Nairobi, Kenya; 13 Nuffield Department of Medicine, University of Oxford, Oxford, UK

**Keywords:** Stillbirth, Infant, Newborn, Death, Infections, Pregnancy, Bacteraemia

## Abstract

**Background:**

Stillbirths and early neonatal deaths remain common in low- and middle-income countries. Maternal infections are likely contributors, but data are extremely limited. We aimed to describe maternal infections at delivery and their association with perinatal death.

**Methods:**

We investigated maternal infections at delivery in two hospitals in East Africa: in Kilifi County Hospital (2011–2017), Kenya, and Hiwot Fana Comprehensive Specialised Hospital (2019–2020), Ethiopia. We compared 642 mothers delivering stillbirths/newborns dying in the first 24 h after birth (cases) and 855 mothers with newborns surviving *≥* 24 h (controls) from pregnancy cohorts. We tested maternal blood with molecular diagnostic panels (TaqMan Array Cards). In Ethiopia, vagino-rectal swabs and oropharyngeal swabs were also tested, along with conventional microbiological testing (blood cultures for bacteraemia). We tested associations between maternal infection and perinatal death for each site. We did a sensitivity analysis restricted to controls with good pregnancy outcomes in Kenya. Where appropriate, we calculated the population attributable fraction.

**Results:**

Maternal bacteraemia was associated with increased odds of perinatal death in Ethiopia (adjusted odds ratio aOR3.7 [1.5–9.2]) in hospital deliveries. Maternal bacterial infection (detected molecularly) was associated with perinatal death in Kenya (aOR2.7 [1.2-6.0]), but only in analyses with controls restricted to those with good pregnancy outcomes. From these analyses, for perinatal deaths among hospital deliveries, the PAF for maternal bacterial infection was 6.1% (4.0%-8.2%) in HFCSH and 4.9% (2.6%-7.2%) in KCH. The study was under-powered to identify specific infections.

**Conclusions:**

Maternal bacterial infection is associated with perinatal death in high-burden settings, and accounted for around 5% of perinatal deaths in hospital deliveries. Improved prevention, and/or detection and treatment of maternal bacterial infection may contribute to reductions in perinatal mortality in hospital deliveries.

**Supplementary Information:**

The online version contains supplementary material available at 10.1186/s12884-026-09068-3.

## Background

Worldwide, deaths in children aged under five years have decreased [1], however, neonatal deaths (days0-27) have decreased more slowly; 2.3 million neonates die each year [1], and a third of these deaths occur on the day of birth [2]. In addition, there were an estimated 1.9 million stillbirths (babies delivered with no signs of life *≥* 28 weeks’ gestation) in 2021 [1]. Reducing deaths at, or just after birth, is thus critical in high burden settings, and nearly a half of all neonatal deaths and stillbirths were in sub-Saharan Africa [1]. Infection is thought to cause a quarter of all neonatal deaths [3] and, although data are limited, accounts for approximately half of all stillbirths [4].

Whilst aetiologic studies in children have been undertaken, data on maternal infections, including those associated with perinatal deaths, are very scarce. The recent WHO Global Maternal Sepsis Study (GLOSS), highlighted the frequency of symptomatic maternal infection, especially in low- and middle-income countries [5], with systematic screening to ascertain clinically-defined cases of possible infection. However, the study was limited in that it only reported infectious aetiologies detected during routine investigation, rather than testing systematically for potential infections [6]. Similarly, whilst multi-country trials of prophylactic azithromycin administration in pregnancy suggest improved outcomes [7], which specific infections were being treated was unclear.

Despite the lack of systematic assessment and testing, from studies focussing on specific infections, we know there are four ways maternal infections in pregnancy can affect the fetus. Firstly, haematogenous infections can spread across the placenta. Examples include *Treponema pallidum* [8], and *Brucella sp* [9], cytomegalovirus [10] and Zika [11]. Secondly, maternal infections can result in placental insufficiency, for example malaria due to *Plasmodium falciparum* [12]. Thirdly, maternal genitourinary tract infection may ascend to the uterus, for example *Escherichia coli* and group B streptococcus (GBS). These infections are leading bacterial causes of perinatal death [13], and common causes of maternal sepsis in high-income countries, such as the UK [14]. Fourthly, systemic disease in the mother, including bacterial sepsis or severe viral infections, such as influenza, dengue [15] and chikungunya [16], are associated with fetal death through severe maternal illness which can, for example, compromise fetal-placental circulation.

We therefore aimed to determine whether maternal infections at delivery were associated with stillbirth or early neonatal death (within 24 h of birth), in high burden settings in East Africa. We considered deaths just before and just after birth to reflect the very high burden at this time, and common aetiologies [17]. In our approach, we wanted to learn from studies undertaken in children, which emphasise the importance of systematic case ascertainment and testing using both conventional microbiological methods (despite low sensitivity [18]), and molecular methods [19–21]. Our objective was to systematically test for maternal infection in case-control studies of mothers, nested within pregnancy cohorts, in two settings in East Africa: Kilifi County Hospital, Kenya and Hiwot Fana Comprehensive Specialized Hospital, Harar, Ethiopia. In Kenya, the approach was retrospective, testing stored samples of maternal blood with multi-pathogen panels, and in Ethiopia, it was prospective, allowing for both conventional and multi-pathogen panel testing on a range of samples (maternal blood, vagino-rectal swabs (VRS) and oropharyngeal swabs (OPS)).

## Methods

### Study design and sites

In Kilifi County Hospital (KCH), a rural county hospital in coastal Kenya, with comprehensive obstetric care and 3-4000 deliveries per year, mothers were recruited at admission for delivery to an observational cohort study (2011 to 2017). Maternal data on the admission and newborn outcomes, were recorded by nurses in a structured maternity record. Maternal blood was routinely collected on admission (using an aseptic technique) and stored at -80 °C [22]. A similar prospective observational cohort study was established in Haramaya University Hiwot Fana Comprehensive Specialized Hospital (HFCSH, 2019–2020), a referral hospital in Harar, Eastern Ethiopia, with comprehensive obstetric care and 4-6000 deliveries per year. Here, maternal blood was collected on admission for delivery (using an aseptic technique) and taken to Hararghe Health Research (HHR) laboratory for bacterial culture (to detect bacteraemia), as well as storage, along with oropharyngeal swabs (OPS), and vagino-rectal swabs (VRS). The HHR laboratory also performed microbiological tests for Child Health and Mortality Prevention Surveillance [23], which ran concurrently with this study, investigating child deaths using minimally invasive tissue sampling (post-mortem). The studies were separate but cases could be in both studies.

Within these cohorts, we nested case-control studies to identify and test associations of different maternal infections with perinatal death. Cases were mothers whose pregnancy yielded a stillbirth (*≥* 28 weeks’ gestation or > 1000 g when the gestational age was unknown) or very early neonatal death (< 24 h following birth). Controls were mothers whose pregnancy yielded a liveborn child (*≥* 28 weeks’ gestation or > 1000 g, and surviving > 24 h after birth). Cases and controls were selected from sampling frames, using random number generation. Where samples had not been taken from those selected, additional participants were randomly selected to achieve the target sample size. Women with multiple deliveries were not excluded, but if any delivery met the case definition, they were included as a case. The study design is summarised in Supplementary Tables 1 and Supplementary Fig. 1, and adherence to STROBE case-control reporting guidelines is shown in Supplementary Table 2 [24].

### Clinical maternal and newborn data collection

Examination of mothers on admission included routine vital signs, as well as symphyseal-fundal height, cervical assessment and general examination. Maternal danger signs at triage included: airway not patent, respiratory rate > 30 or < 10 breaths/minute, systolic blood pressure > 160 or < 90 mmHg, diastolic blood pressure > 90 mmHg, heart rate < 40 or > 120 beats/minute, unconscious or alert only to pain, and other obstetric emergencies (including imminent delivery) requiring immediate intervention. Maternal and newborn outcomes at delivery were recorded, including sex, and birthweight (using SECA digital scales).

### Laboratory analyses

We used molecular testing on multi pathogen panels in both sites to test samples for bacterial, viral, parasitic and fungal infections (Supplementary Table 3 details species-specific molecular targets). In both sites we tested maternal blood, and in HFCSH we also used molecular methods to test vagino-rectal and oropharyngeal swab samples. Different TAC cards were used for each specimen type. In both sites, detection of infections from the extracted nucleic acid was done using TaqMan array cards (TAC), amplifying using QuantStudio 7 Flex Real-Time PCR System (Applied Biosystems/Singapore**)**. Analysis was done with CDC QuantStudio software; QS7 v1.2 CDC. These methods were developed to support Child Health and Mortality Prevention Surveillance, and were optimised for molecular testing and quality assurance [25].

In HFCSH, we also used conventional microbiological methods to detect maternal bacteraemia and test vagino-rectal swabs. For blood cultures, BACTEC (Becton Dickinson, South Africa) bottles were incubated at 37 ± 2 °C in BACT/ALERT^®^ 3D Microbial Detection Systems (BioMérieux/USA) for five days; bottles alerting positive were Gram-stained and sub-cultured as appropriate. Frozen, stored vagino-rectal swabs were also thawed and sub-cultured as appropriate. Pathogens were identified by morphological appearance, Gram staining and standard biochemical tests.

### Statistical analyses

We aimed to identify pathogens strongly associated (OR > 3) with perinatal death where the prevalence of infection in controls was low (~ 1%), (Supplementary Table 4). We increased the proportion of controls to cases in Kenya to enable a sensitivity analysis including only controls with good pregnancy outcomes; those whose babies were born by a spontaneous vaginal delivery (cephalic), had a birthweight *≥* 2500 g, and did not require admission to the neonatal unit. We calculated the odds ratios detected (2 to 8) based on varying prevalence of exposure in controls (0.001 to 0.1) and selected a sample size of 350 cases in each site, 350 controls in HFCSH, 500 controls in KCH.

We described maternal participants in the study from each site, in terms of socio-demographics, and signs and symptoms of infection. All statistical analyses were conducted in Stata release 14 (StataCorp, College Station, TX). We tested the association of each maternal characteristic with perinatal death using logistic regression. We included maternal age in the final model as an a priori confounder. We used a similar approach for the characteristics of the participants’ babies, where we described birth weight and sex, and used logistic regression to test the association with perinatal death. We included the sex of the baby in the final model as an a priori confounder. For both models we tested characteristics (*p* < 0.1) in a multivariable model and down-selected based on p values (*p* < 0.1) for the final model. For maternal participants and their babies, we did these analyses for each site separately, and combined, to increase power, setting site as a random effect. We repeated these analyses using multiple imputation to assess impact of missing data, imputing with reference to other variables in the dataset (including site) and by case/control status (site was set as a random effect in subsequent analyses). We also did an exploratory analysis to test the association of signs and symptoms of infection with maternal bacteraemia.

For infections in maternal blood, we described TAC detections for bacteria, viruses, parasite and fungi, and blood culture results for clinically significant maternal bacteraemia. We excluded cultures of likely contaminants (coagulase negative staphylococcus and Bacillus spp.) from this definition, but included these participants in analysis. We tested the association of clinically significant maternal bacteraemia with perinatal death using logistic regression; we also tested the associations of species-specific bacteraemia with perinatal death. We similarly tested the association of all bacteria detected by TAC, all viruses detected by TAC, and each individual target (bacteria, virus, parasite or fungus) detected by TAC, with perinatal death. We did this for each site separately and then combined (to increase power), specifying site as a random effect. Similarly, we also tested the association of the combination of bacterial targets detected either with TAC or blood culture with perinatal death for both sites combined (to increase power), specifying site as a random effect. Where numbers of detections, or bacterial isolates were small, we used Fisher’s exact test to assess associations. We ran a sensitivity analysis using KCH data, restricting the control group to participants with good pregnancy outcomes, to check whether including controls with serious (but non-fatal) infections had biased our main results toward the null. We did additional analyses for HFCSH with analysis of infections detected using TAC on maternal oropharyngeal swabs (OPS), and maternal vagino-rectal swabs (VRS). We also described conventional microbiological culture results from VRS in HFCSH. We tested the association of each target detected with perinatal death and each positive culture with perinatal death using logistic regression for HFCSH.

For results where infections were associated with perinatal death (*p* < 0.1) we used multivariable models to adjust for potential confounders (maternal age, education level, marital status, nulliparity, baby/fetal sex) and down-selected based on p values (*p* < 0.1) for the final model, including maternal age and baby/fetal sex as a priori confounders. We repeated these analyses using multiple imputation to assess impact of missing data as described above. As an exploratory analysis, intended to highlight important infections for future study, we did not make a formal correction for the potential for chance with the use of multiple statistical tests.

We calculated the population attributable fraction (PAF) for results where infections were associated with perinatal death and there was good evidence for causality, based on assessment using the Bradford-Hill criteria. PAFs were calculated using the punafcc command in Stata release 14, based on maximum likelihood estimation of the attributable fraction from multivariable logistic models [26].

We identified cases which were also included in CHAMPS [23], to compare findings from maternal investigation in this study and stillbirth/neonatal post-mortem investigations in that study that were used to determine cause of death.

## Results

### Participants

**Fig. 1:**
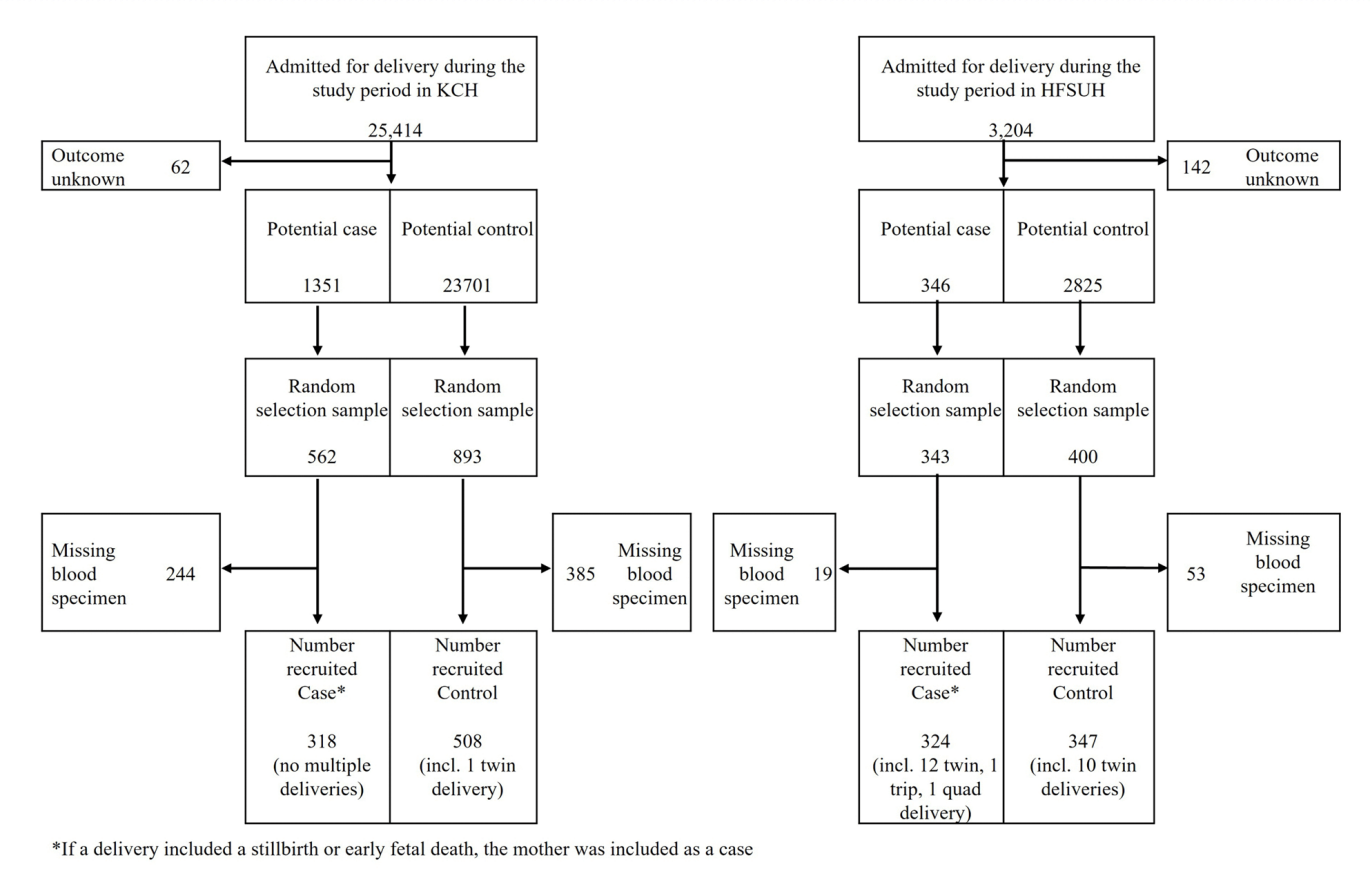
Recruitment of cases and controls from among pregnant women delivering in Kilifi County Hospital, Kenya (1 Jan 2011-5 Jun 2017) and Hiwot Fana Comprenhensive Specialised Hospital, Ethiopia (22 May 2019-5 Sep 2020)

In KCH 25,414 pregnant women attended the labour ward between 1 Jan 2011 and 5 June 2017. In HFCSH, 3204 pregnant women attended the labour ward between 22 May 2019 and 5 September 2020. Following randomisation, in the final analysis, there were 318 cases and 508 controls from KCH and 324 cases and 347 controls from HFCSH (Fig. 1).

In both KCH and HFCSH, a majority of the women were married and multiparous (Table 1). The majority of women were educated to primary level in KCH (59% of participants in the control group), but not in HFCSH (26% of controls). Signs and symptoms of infection were infrequent, but women commonly presented with an emergency sign (89% and 49% of participants in control group, for KCH and HFCSH respectively). In the combined analysis across sites, socio-demographic and clinical signs associated with perinatal death included no education (adjusted odds ratio aOR1.9 [1.5–2.5]), and at least one emergency sign at delivery (aOR1.9 [1.5–2.4]). Among the characteristics of babies, low and very low birthweight were associated with perinatal death (aOR3.7 [2.8–4.9] and aOR18.3 [10.2–32.5], respectively). Site-specific analyses are detailed in Supplementary Tables 5 and 6, combined in Supplementary Table 7. Estimates of effect (odds ratios) for characteristics associated with perinatal death were comparable when multivariable analyses were repeated with imputation (data not shown). In the exploratory analysis, a history of fever (aOR6.1 [95% CI: 1.1–33.0]) and at least one emergency sign at delivery (OR2.1 [1.1–5.1]) were associated with maternal bacteraemia (Supplementary Table 8) in HFCSH.

**Table 1:** Characteristics of mothers and babies in the study, by case and control status, for Kilifi County Hospital, Kenya and Hiwot Fana Comprehensive Specialised Hospital, Ethiopia

Maternal characteristic	Kilifi County Hospital				Hiwot Fana Comprehensive Specialised Hospital			
	Cases *N* = 318	(%)	Controls *N* = 508	(%)	Cases *N* = 324	(%)	Controls *N* = 347	(%)
Age, years								
< 20	43	13.5	96	18.9	33	10.2	30	8.6
20 to < 30	157	49.4	277	54.5	165	50.9	218	62.8
30 to < 40	98	30.8	124	24.2	97	29.9	68	19.6
* ≥* 40years	15	4.7	10	2.0	5	1.5	10	2.9
Missing^b^	24	7.5	21	4.1	24	7.4	21	6.1
Marital status								
Married	278	87.4	457	90.0	305	94.1	321	92.5
Single	26	8.2	43	8.5	1	0.3	1	0.3
Divorced	3	0.9	2	0.4	0	0.0	0	0.0
Widowed	0	0.0	0	0.0	0	0.0	0	0.0
Missing^b^	11	3.5	6	1.2	18	5.6	25	7.2
Education level								
None	64	20.1	73	14.4	219	67.6	135	38.9
Primary	181	56.9	299	58.9	38	11.7	91	26.2
Secondary	55	17.3	88	17.3	16	4.9	51	14.7
Higher	7	2.2	33	6.5	24	7.4	36	10.4
Missing^b^	11	3.5	15	3.0	27	8.3	34	9.8
Nulliparous								
No	209	65.7	322	63.4	205	63.3	219	63.1
Yes	102	32.1	174	34.3	90	27.8	100	28.8
Missing^b^	7	2.2	12	2.4	29	9.0	28	8.1
History of fever								
No	305	95.9	498	98.0	290	89.5	320	92.2
Yes	8	2.5	5	1.0	3	0.9	4	1.2
Missing^b^	5	1.6	5	1.0	31	9.6	23	6.6
Prelabour Rupture of Membranes > 18 h								
No	268	84.3	438	86.2	256	79.0	271	78.1
Yes	15	4.7	24	4.7	38	11.7	44	12.7
Missing^b^	35	11.0	46	9.1	30	9.3	32	9.2
Dysuria								
No	309	97.2	488	96.1	291	89.8	323	93.1
Yes	3	0.9	15	3.0	1	0.3	2	0.6
Missing^b^	6	1.9	5	1.0	32	9.9	22	6.3
Positive nitrite and/or leucocyte								
No	216	67.9	344	67.7	103	31.8	125	36.0
Yes	61	19.2	117	23.0	41	12.7	52	15.0
Missing^b^	41	12.9	47	9.3	180	55.6	170	49.0
Emergency signs^a^								
No	231	72.6	451	88.8	111	34.3	170	49.0
Yes	87	27.4	57	11.2	213	65.7	177	51.0
Mode of delivery								
Vaginal	249	78.3	400	78.7	250	77.2	257	74.1
Caesarean section	60	18.9	96	18.9	74	22.8	90	25.9
Missing^b^	9	2.8	12	2.4	0	0.0	0	0.0

**Baby characteristics**	**N =318**	**%**	**N =509**	**%**	**N= 338**	%	**N =356**	**%**
Birthweight (g)								
1000-1500	56	17.6	8	1.6	58	17.2	7	2.0
1500- 2500	88	27.7	81	15.9	87	25.7	32	9.0
Over 2500	147	46.2	412	80.9	142	42.0	291	81.7
Missing^b^	27	8.5	8	1.6	51	15.1	26	7.3
Sex								
Male	150	47.2	227	44.6	177	52.4	176	49.4
Female	144	45.3	271	53.2	124	36.7	151	42.4
Missing**	24	7.5	11	2.2	37	10.9	29	8.1

^a^Maternal danger signs included any of the following airway not patent, respiratory rate >30 or <10 breaths/minute, systolic blood pressure >160 or <90 mmHg, diastolic blood pressure >90 mmHg, heart rate <40 or >120 beats/minute, unconscious or alert only to pain, and other obstetric emergencies (including imminent delivery) requiring immediate intervention

^b^Clinical data were collected as part of routine care, by clinical staff in health care facilities. In high-burden settings infrastructure and resources are limited, and the missing data reflects real-world challenges in observational studies in such settings

### Detection of infection and association with perinatal death/stillbirth

In HFCSH, maternal bacteraemia occurred in 24/324 (7.4%) cases and 10/347 (2.9%) controls (OR 2.7 [1.3–5.7], aOR3.7 [1.5–9.2], Supplementary Table 9). In KCH, in our sensitivity analysis including only mothers with good pregnancy outcomes as controls, bacterial detection in maternal blood with TAC was associated with perinatal death (24/318 (7.5%) cases, 10/315 (3.2%) controls, OR2.5 [1.2–5.3], aOR2.7 [1.2-6.0], Supplementary Table 10). We did not find evidence of an association between maternal blood bacterial detection by TAC and perinatal death with regular controls, at either site, or at both sites combined.

**Fig. 2:**
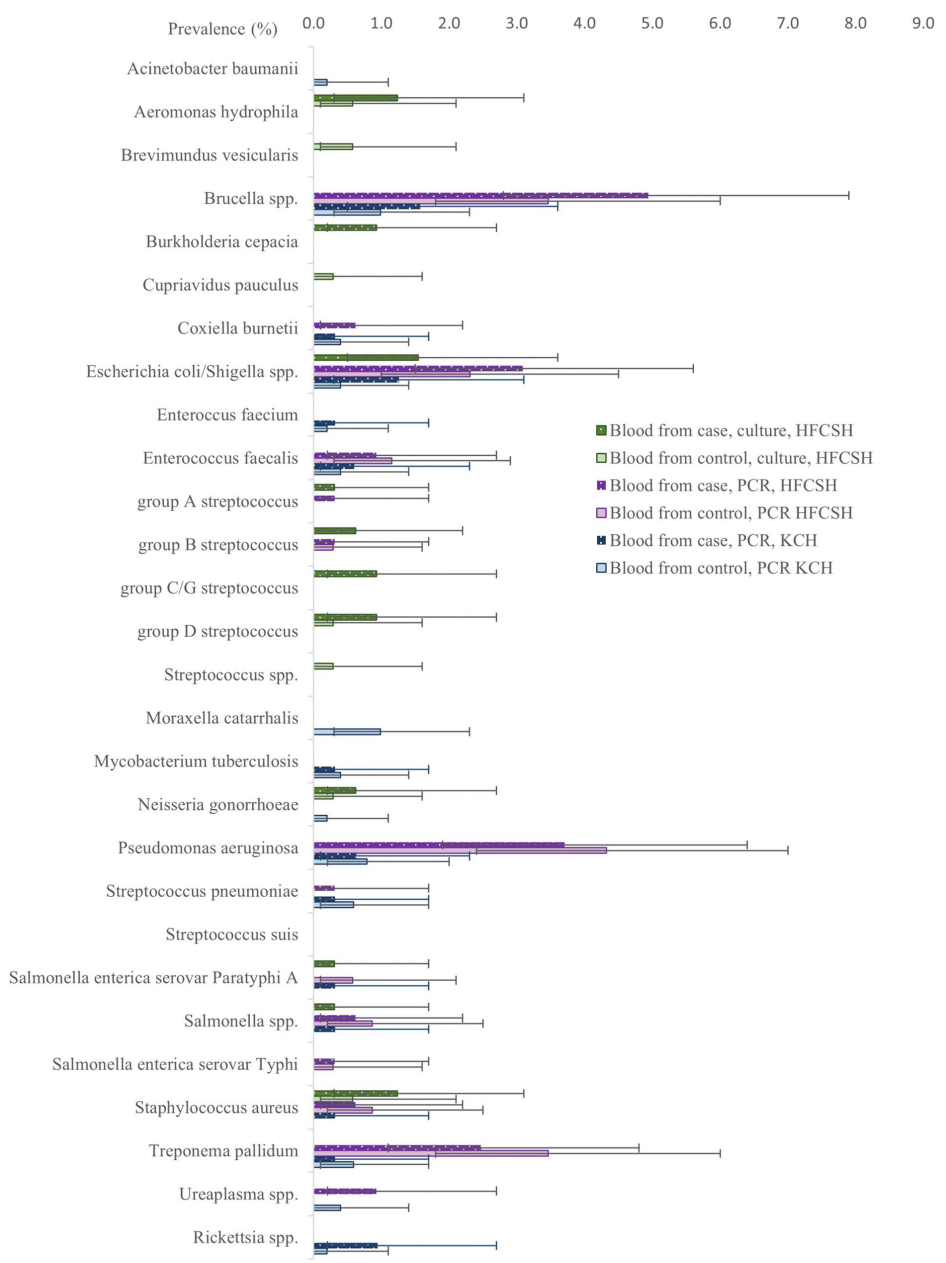
Prevalence of bacterial species, detected by PCR in blood in maternal participants in Kilifi County Hospital (KCH), Kenya, and prevalence of bacterial species detected by PCR or culture in blood, in maternal participants in Hiwot Fana Comprehensive Specialised Hospital (HFCSH), Ethiopia. Error bars show 95% confidence interval

For specific pathogens (Fig. 2), only *Escherichia coli* bacteraemia was associated with perinatal death (Fisher’s exact test *p* = 0.026). However, maternal bacteraemia with *Escherichia coli* (*n* = 5), *Streptococcus agalactiae* (*n* = 2), *Streptococcus pyogenes* (*n* = 1), Streptococcus Group C&G (*n* = 3), *Burkholderia cepacia* (*n* = 3) and *Salmonella paratyphi* (*n* = 1), whilst rare, occurred only in cases. In the combined analysis of maternal blood cultures and maternal blood bacterial DNA detection using TAC, *E. coli* culture and/or *E. coli*/*Shigella spp.* detection was associated with perinatal death across sites (18/642 (2.8%) cases, 10/855 (1.2%) controls OR 2.2[1.0-4.8], aOR2.6 [1.1–6.3], Supplementary Table 11). *Treponema pallidum* was not associated with perinatal death (OR0.7 [0.3–1.6], for both sites combined). Detailed results for maternal bacteraemia, maternal blood target detection and association with perinatal death for HFCSH, KCH and the two sites combined are in Supplementary Tables 12–15. Details of the sensitivity analysis results are in Supplementary Table 16, Supplementary Fig. 2, and details of the maternal blood targets detected by PCR and/or cultured combined across both sites in Supplementary Table 17.

**Fig. 3:**
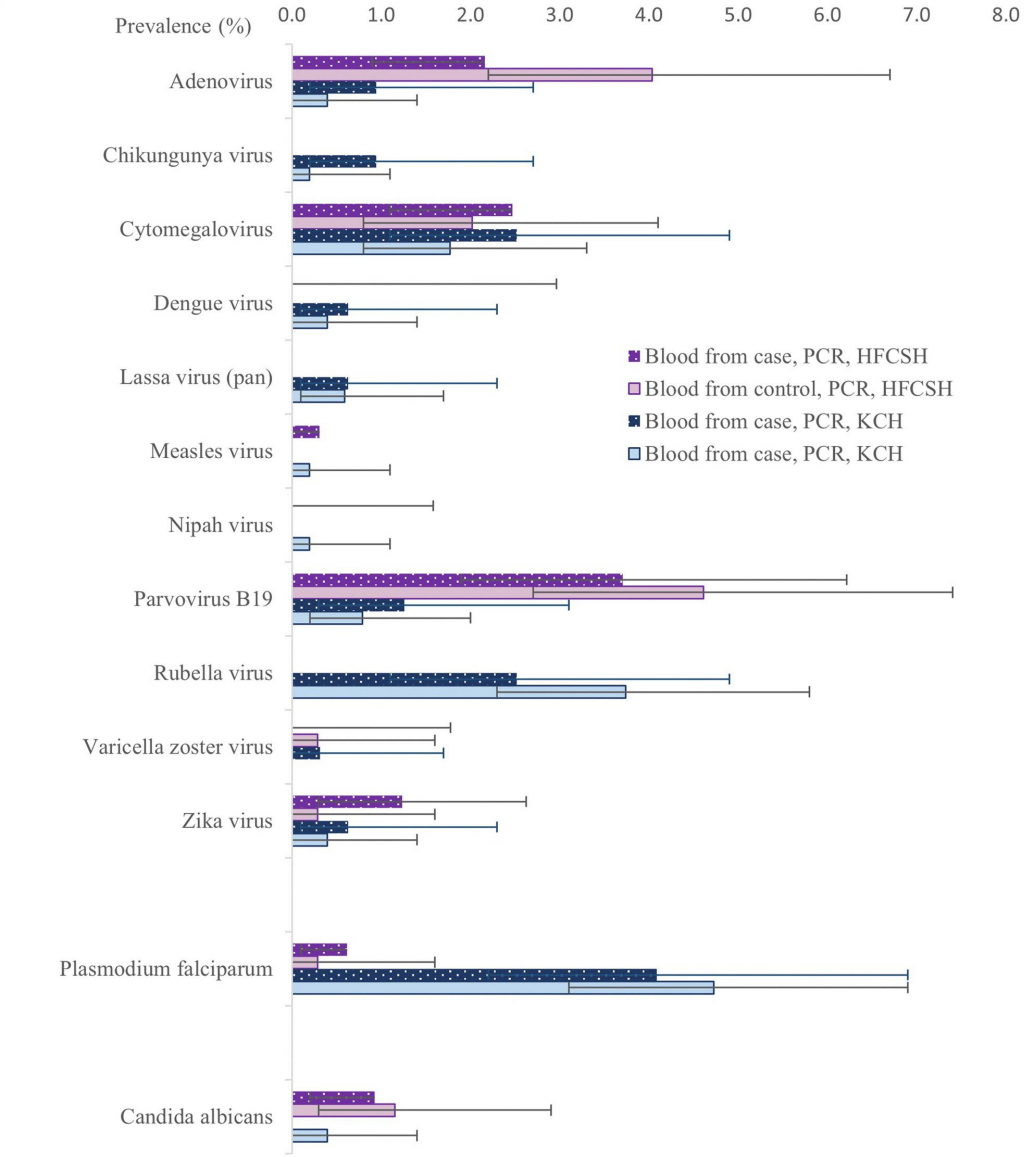
Prevalence of viral, parasitic and fungal species, detected by PCR in blood in maternal participants in Kilifi County Hospital (KCH), Kenya and maternal participants in Hiwot Fana Comprehensive Specialised Hospital (HFCSH), Ethiopia. Error bars show 95% confidence intervals

We did not find evidence of an association between viral targets in maternal blood detected by TAC, individually or combined, and perinatal death. Viral targets commonly detected in maternal blood were cytomegalovirus and parvovirus B-19 in both sites (1.9% and 2.3% of participants in control group respectively), adenovirus in HFCSH (4.0% controls) and rubella at KCH (3.7% controls). Zika virus was detected, at low levels at both sites (0.4% controls) but other *Aedes sp*. mosquito-borne viruses, e.g. chikungunya and dengue viruses (0.2% and 0.4% controls, respectively), were only detected in KCH (Fig. 3 and Supplementary Tables 13–15).

We did not find an association between molecular detection in maternal blood of any individual parasitic or fungal targets and perinatal death. The only parasite detected was *Plasmodium falciparum*, which was common in KCH (4.7% controls) but was not associated with perinatal death (OR 0.9 [0.5–1.8]), Fig. 3 and Supplementary Tables 13–15). The only fungus detected was *Candida albicans*, which was more common in HFCSH (2.4% controls).

**Fig. 4:**
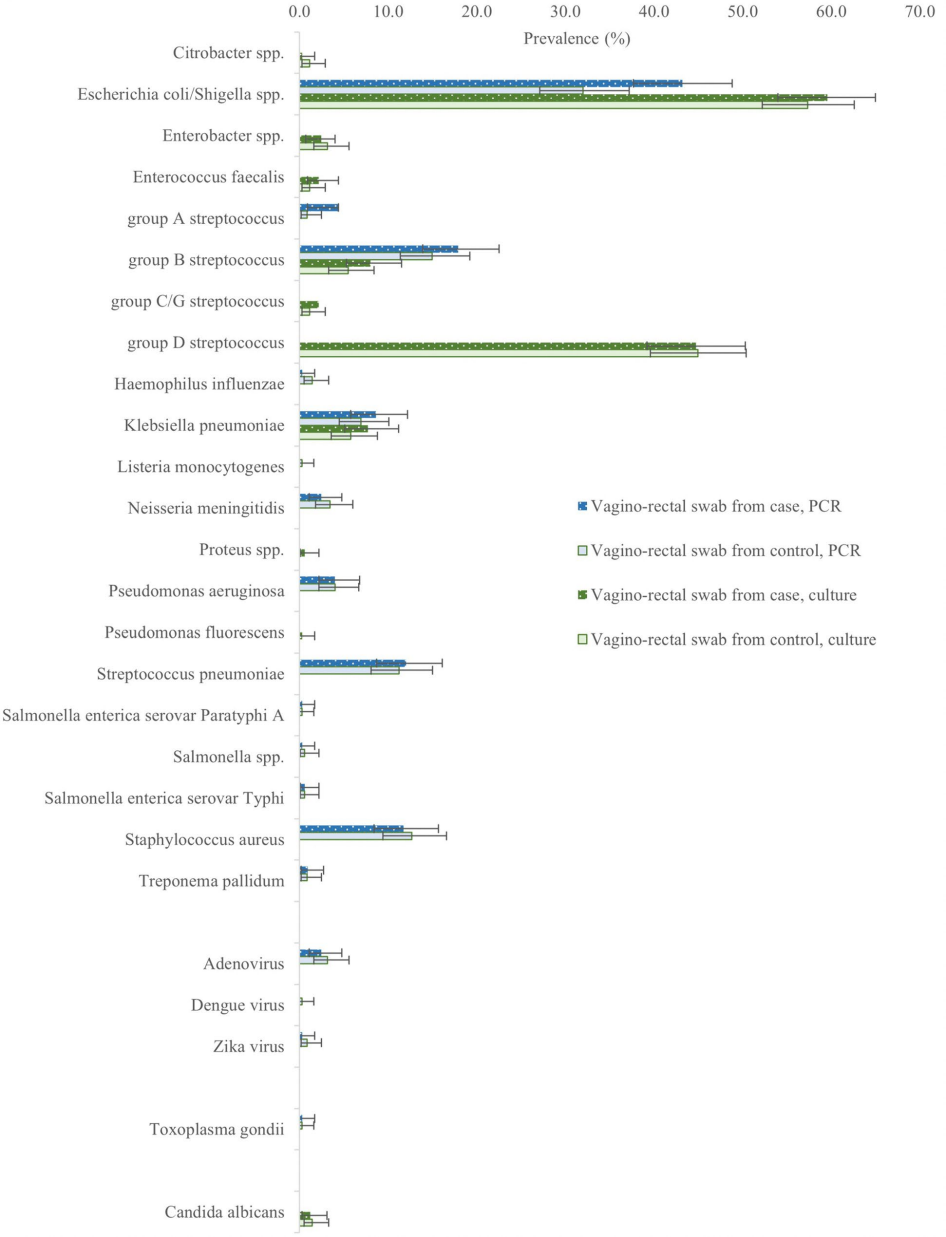
Prevalence of bacterial, viral, parastic or fungal species detected by PCR or bacterial culture from vagino-rectal swabs in maternal participants in Hiwot Fana Comprehensive Specialised Hospital, Ethiopia. Error bars show 95% confidence intervals

For maternal vaginal-rectal swab (VRS) samples, we found some evidence of an association between molecular detection of *E. coli/Shigella spp.* with perinatal death (140/324 (43%) cases, 111/347 (32%) controls OR1.6 [1.2–2.2]; adjOR1.3 [0.9–2.9], Supplementary Tables 18, 20). However, we found no association between any individual conventional bacterial culture from VRS and perinatal death (Supplementary Table 19). Group D Streptococcus, cultured in 45% of controls, was not included in the TAC panel (Fig. 4).

**Fig. 5:**
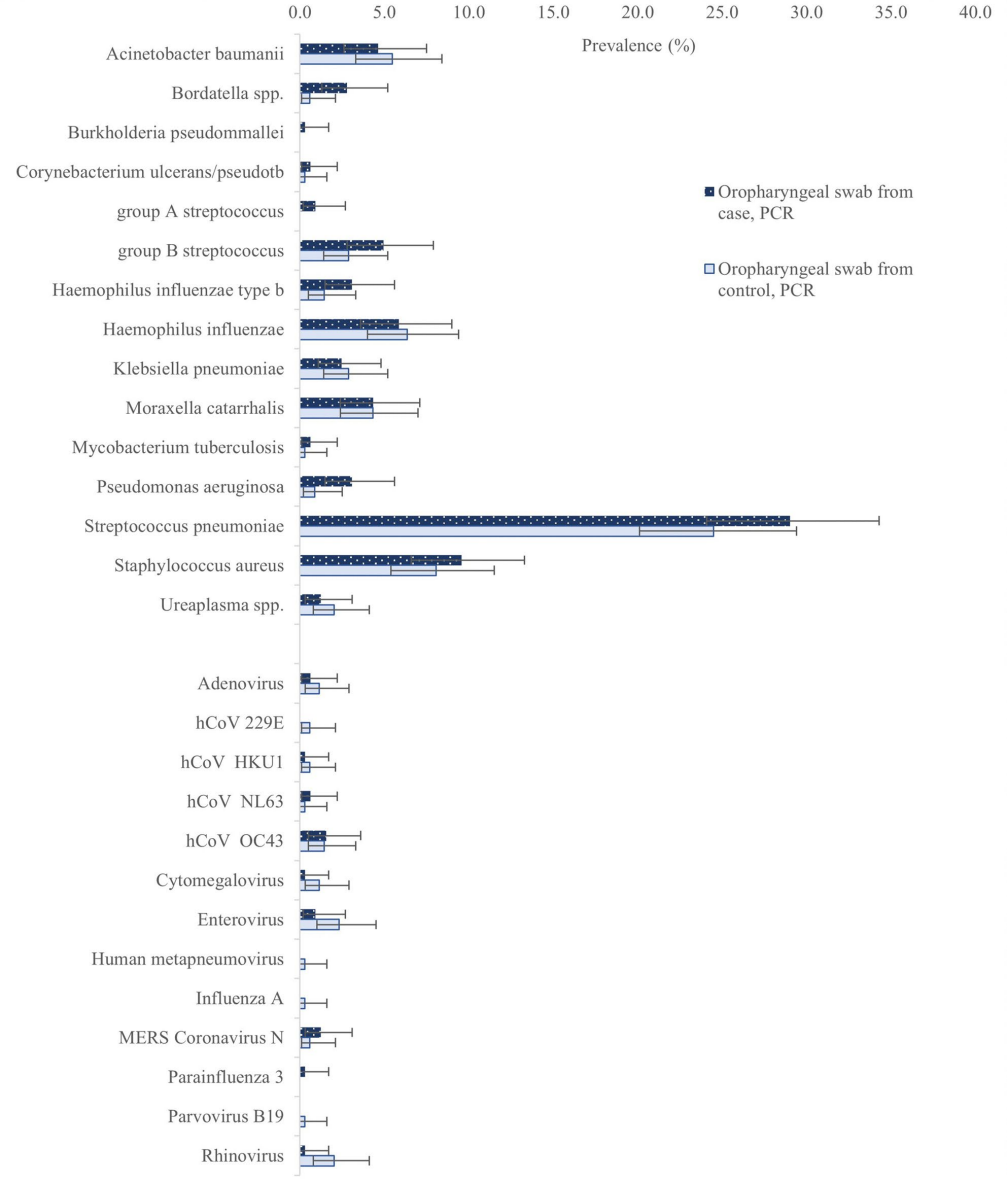
Prevalence of bacterial and viral species detected by PCR in oropharyngeal swabs from maternal participants in Hiwot Fana Comprehensive Specialised Hospital, Ethiopia. Error bars show 95% confidence interval

For oropharyngeal swab (OPS) samples, we found that molecular detection of *Bordetella spp.* (pertussis or parapertussis) was associated with perinatal death (9/324 (2.8%) cases, 2/347 (0.6%) controls, OR4.9[1.1–23.0]). Multivariable analyses only included Bordetella spp. in cases (missing data), so aORs are not reported. We did not find an association between molecular detection on OPS of any other individual bacterial, viral or fungal target and perinatal death (Supplementary Tables 21–22). The bacterial target detected most frequently was *Streptococcus pneumoniae* (24.5% controls, Fig. 5). Estimates of effect (odds ratios) for infections associated with perinatal death were repeated with multiple imputation, and comparable to the unadjusted estimate of effect for *Bordetella spp.* (data not shown).

### Estimates of the population attributable fraction (PAF)

Maternal bacteraemia in HFCSH was moderate to strongly associated with perinatal death, and sensitivity analysis of maternal bacterial infection in KCH was consistent with this. The associations were specific to bacteria, fulfilling the causal criteria of strength, consistency and specificity, as defined by Bradford Hill. The association was biologically plausible, as bacteria are known to cause ascending infection around delivery, and there was also temporality in the association, fulfilling plausibility and temporality criteria. In our study we did not establish a biological gradient, coherence, experiment or analogy, but concluded that with several Bradford Hill criteria met, that there was sufficient evidence that the association between maternal bacterial infection in blood and perinatal death was likely to be causal, to warrant calculation of the population attributable fraction. The PAF of maternal bacteraemia, in participants in HFCSH, to perinatal death was 6.1% (4.0%-8.2%), and for maternal bacterial infection in participants in KCH (sensitivity analysis) was 4.9% (2.6%-7.2%).

### Comparison with child health and mortality preventions surveillance (CHAMPS)

We identified 14 cases where data were available on the cause of death from CHAMPS [23]. Of these, the underlying cause of stillbirth/early neonatal death was intrauterine hypoxia in 10/14, congenital abnormality in 2/14, intrauterine growth restriction in 1/14 and neonatal sepsis with *E. coli* in a preterm neonate in 1/14. In the neonatal sepsis case we did not detect *E. coli* bacteria in maternal blood (TAC or culture), but *E coli/Shigella* was detected using TAC from VRS, and *E. coli* was cultured from VRS. Conversely, there were two cases (one recorded as intrauterine hypoxia, one as intrauterine growth restriction) where maternal bacterial infection was cultured in blood (*Burkholderia cepacia*, and *Aeromonas hydrophila/caviae* respectively), and one case (intrauterine hypoxia) where maternal bacterial infection was detected using TAC in blood (*Pseudomonas aeruginosa)*.

## Discussion

Maternal bacterial infections at delivery are associated with perinatal death in high-burden settings in East Africa. *Escherichia coli* is a probable contributor, but other infections are also likely to be important. In our study, around 5% of perinatal deaths in hospital were attributable to maternal bacterial infection at delivery. Improved prevention, and/or detection and treatment of maternal bacterial infection may contribute to reductions in perinatal mortality in hospital deliveries. We did not find evidence associating viral, parasitic or fungal infections at delivery with perinatal death.

Our study was strengthened through the inclusion of two sites with contrasting ecologies, allowing assessment of similarities and differences across sites, and testing multiple sample types, using both conventional and molecular methods. However, there were differences in the time periods during which mothers were recruited into cohorts, and the samples and testing undertaken, which limit comparisons. In analyses, whilst multi-pathogen panels were a strength of the study, we undertook a high number of statistical tests, increasing the probability of detecting associations by chance. Therefore, where results are consistent, and there is a high level of plausibility, we have higher confidence in our findings, such as the overall association between maternal bacterial blood infection and perinatal death. In contrast, we recognise that the evidence here is insufficient to confirm true associations for specific infections. In fact, there were few specific infections (only *E coli*, *Bordetella spp*.) where we found a specific association with perinatal death. Our study was adequately powered to detect strong associations (odds ratios > 3) for exposures present in ~ 1% of controls, but many bloodstream infections were detected at lower prevalences than this, limiting our power for these pathogens. In addition, whilst our study benefited from sensitive molecular methods, the potential decrease in clinical specificity using molecular detection may have increased the probability of a null finding. Inclusion, in the control group, of participants with clinically significant, but non-fatal, infection may also have biased our primary analyses towards the null, as an association between maternal bacterial infection in blood was only found when cases were compared to controls with good pregnancy outcomes.

We found an association between *E. coli* in maternal blood and perinatal death. Whilst recognising the potential role of chance here, it is biologically plausible with ascending infection around birth, and it is consistent with independent cord blood studies of neonatal sepsis at KCH [27]. We also found maternal recto-vaginal colonisation with *E. coli* was associated with perinatal death. This may reflect risk of ascending infection, but there was also evidence of confounding consistent with the observation that *E. coli* vagino-rectal colonisation is more common in lower socio-economic groups [28], who have an increased risk of perinatal death overall. Similarly, the association between *Bordetella spp*. and perinatal death may be true, but it may also be due to chance, or confounding; poor uptake of whole cell infant pertussis vaccination in the community (and thus potential increased risk of maternal infection) is independently associated with poverty and/or lack of access to health care. Our findings in relation to *E coli* recto-vaginal colonisation and *Bordetella spp* in the oropharynx are also limited in that these samples were only taken in Ethiopia and consistency across sites cannot be determined.

Some infections were sufficiently prevalent in controls (~ 2% in blood), to provide the study with adequate power to detect a moderate association (odds ratios > 2.5) with perinatal death. This applied to *Brucella spp.*, *Pseudomonas aeruginosa* and *Treponema pallidum*, adenovirus, CMV, parvovirus B19, rubella and to *Plasmodium falciparum*. However, we did not find good evidence of associations between these infections and perinatal mortality. For infections known to cause adverse perinatal outcomes in LMICs, which are the target of preventive programs, such as syphilis (caused by *Treponema pallidum*) and malaria (caused by *Plasmodium falciparum*) this is potentially important, but it does not exclude causal association with perinatal death, or contribution to other adverse perinatal outcomes, such as low birth weight, or congenital syphilis, with attendant long-term sequelae. These and other infections (particularly viruses, for example CMV, Parvovirus B-19 and rubella) can have a lag between infection and adverse perinatal outcome and so associations would not be detected in our study design. Some infections may also be more likely to result in spontaneous abortion or stillbirth, than early neonatal death. Combining stillbirths with early neonatal deaths may also have decreased power to detect associations. In contrast, ascending bacterial infections (such as *E. coli* or group B streptococcus), are likely to act at, and around, delivery, and are found in both stillbirths and early neonatal deaths [23], making them more likely to be detected in our study design. We also note the absence of *Klebsiella pneumoniae* in maternal blood, despite increasing reports of is contribution to neonatal sepsis in high burden settings [13, 23]. This likely reflects the differing pathogenesis of *Klebsiella pneumoniae*, acquired postnatally, frequently in preterm neonates admitted to hospital, compared to the ascending infection more frequent for *E. coli* or group B streptococcus infections.

Our findings suggest around 5% of the perinatal deaths in these hospitals were attributable to maternal bacterial infection at delivery. There was consistency across sites, despite differences, for example, in maternal education levels and the range of infections across our sites, implying a degree of generalisability. Our findings are not, however, generalisable to lower levels of health care, or home deliveries; pregnant women delivering in hospitals are a biased sample, often referred with complications, and they may have had intrapartum antibiotics. Whether the perinatal deaths we attribute to maternal bacterial infection are already being attributed to infection in the child, rather than the mother, is difficult to distinguish. In the 14 cases where we were able to compare investigations of both the mother and baby there was no evidence of infection in the three perinatal deaths born to mothers with evidence of bacterial infection in blood. Conversely, one of the 14 babies experienced early neonatal death with evidence of *E. coli* infection, but we did not detect *E. coli* in maternal blood.

## Conclusions

Overall, our study indicates an important association between bacterial infections of maternal blood at delivery, and perinatal death. In hospital-based deliveries in East Africa, maternal bacterial blood infection may account for around 5% of perinatal deaths. Our study was underpowered for specific infections, and a much larger study would be required to test associations for specific infections with perinatal death. Ideally this would be a nested case-control study within a community setting, to reflect the population. Sampling maternal blood earlier in pregnancy may be needed to find true associations for infections which have the highest risk in the first, or early second, trimester of pregnancy, as well as at delivery. Improving our understanding of the aetiology of maternal infection, through studies, but also through maternal health programs and improved diagnostics, is important to inform interventions and reduce the burden of death at birth.

## Supplementary Information

Supplementary Material 1.

## Data Availability

The datasets generated and/or analysed during the current study are not publicly available due to ethical and legal restrictions. Data may be made available from the corresponding author upon reasonable request, subject to appropriate approvals.
